# Clinical correlates of nocardiosis

**DOI:** 10.1038/s41598-020-71214-4

**Published:** 2020-08-31

**Authors:** Ili Margalit, Elad Goldberg, Yaara Ben Ari, Haim Ben-Zvi, Yael Shostak, Ilan Krause, Khitam Muhsen

**Affiliations:** 1 Department of Internal Medicine F-Recanati, Rabin Medical Center, Beilinson Hospital, Petah Tikva, Israel; 2 Sackler Faculty of Medicine, Tel Aviv University, Ramat Aviv, Tel Aviv, Israel; 3 Microbiology Laboratory, Rabin Medical Center, Beilinson Hospital, Petah Tikva, Israel; 4 Pulmonary Institute and Department of Internal Medicine D, Rabin Medical Center, Beilinson Hospital, Petah Tikva, Israel; 5 Department of Epidemiology and Preventive Medicine, Sackler Faculty of Medicine, School of Public Health, Tel Aviv University, Ramat Aviv, Tel Aviv, Israel

**Keywords:** Bacterial infection, Risk factors, Epidemiology

## Abstract

*Nocardia* is an opportunistic pathogen that most frequently affects the lungs. Evidence is limited regarding the risk factors for nocardiosis. The current study assessed clinical correlates of nocardiosis. A retrospective study was conducted based on medical records of consecutive adult patients (N = 60) with nocardiosis hospitalized during 2007–2018 at a tertiary hospital in central Israel. A matched comparison group of 120 patients was randomly selected among hospitalized patients with community-acquired pneumonia. Multivariable conditional logistic regression models were fitted. Immunosuppressive pharmacotherapy was positively associated with nocardiosis (matched odds ratio [OR] 4.40, 95% confidence interval [CI] 2.25–8.62, *p* < 0.001), particularly corticosteroid therapy (matched OR 4.69, 95% CI 2.45–8.99, *p* < 0.001). Systemic corticosteroid therapy was strongly associated with pulmonary nocardiosis (matched OR 5.90, 95% CI 2.75–12.66, *p* < 0.001). The positive association between solid organ transplantation and nocardiosis was attenuated following adjustment for systemic corticosteroids in a multivariable model. The association between corticosteroid therapy and nocardiosis appeared stronger in patients with chronic pulmonary disease (OR 5.74, 95% CI 2.75–12.66, *p* < 0.001) than in the pooled analysis of all nocardiosis cases. In conclusion, corticosteroid therapy was strongly correlated with nocardiosis, particularly among individuals with chronic pulmonary disease and in pulmonary nocardiosis.

## Introduction

Nocardiosis is an opportunistic infection that typically occurs in individuals with a history of solid organ transplantation (SOT)^1^, malignancy^2^, human immunodeficiency virus infection^3^ or chronic pulmonary diseases^4^. Evidence on the independent contribution of each of these conditions to nocardiosis risk remains limited, since most studies were case-series and lacked a comparison group. It is also unclear if the likelihood of nocardiosis is related to the underlying disease itself or to the immunosuppression therapy administered against it.

Corticosteroid therapy is prevalent amongst individuals diagnosed with nocardiosis^5,6^. A European multicenter case–control study conducted among SOT recipients (SOTR) showed that corticosteroid dosage was related to the risk of nocardiosis (odds ratio [OR] 1.12, 95% confidence interval [CI] 1.03–1.22 for each milligram increase in corticosteroid dose), and a strong positive association was found between high calcineurin inhibitor and nocardiosis (OR 6.11, 95% CI 2.58–14.51)^1^. As corticosteroid therapy is the standard of care for many conditions that might be also associated with nocardiosis, such as SOT, chronic pulmonary diseases and cancer, the independent role of each factor, warrant further investigation.

As *Nocardia* is mostly transmitted by inhalation or aspiration^7^, the lungs are the primary site of infection in more than two-thirds of the nocardiosis cases^3^. However, evidence on the risk factors for nocardiosis, beyond the general propensity to develop bacterial pneumonia, remains unknown.

The current study aimed to assess clinical correlates of nocardiosis, and was designed to identify their contribution to the development of nocardiosis, beyond the risk of pneumonia requiring hospitalization.

## Methods

### Study design and population

A retrospective study was conducted based on the medical records of patients aged 18 years or older who were hospitalized during 2007–2018 at Rabin Medical Center (RMC), a tertiary hospital in central region of Israel. All citizens in Israel have health insurance, according to the universal health insurance law implemented in Israel since 1995. Each citizen is insured by one of the four health maintenance organizations (HMOs) that provide primary/community health-care services and cover hospitalizations costs. The hospitalization records that were the main source of information in this study are linked to the medical records of the primary/community health care system run by the HMOs. This enabled comprehensive assessment of clinical and laboratory features of all the patients included in the study.

We reviewed the microbiology laboratory records for all the patients with *Nocardia* isolation during 2007–2018. *Nocardia* isolation was defined as the laboratory detection of *Nocardia*, sampled from an individual patient. We identified all consecutive patients with *Nocardia* isolation who received a formal diagnosis of nocardiosis by an infectious disease specialist, and who were treated accordingly. The exclusion criterion was *Nocardia* colonization. To ensure appropriate classification, an infectious disease specialist (EG) reviewed the medical charts of all the patients from whom *Nocardia* was isolated, and confirmed the diagnoses, as described elsewhere^8^.

A comparison (control) group included hospitalized patients with a clinical diagnosis of pneumonia during 2007–2018, who were identified by hospitalization diagnoses codes of the World Health Organization International Classification of Diseases 9th Revision (ICD-9)^9^ (see the list of diagnoses in the supplementary methods). The rationale behind the selection of a control group with pneumonia is that nocardiosis mostly affects the lungs, and we aimed to identify specific correlates of nocardiosis beyond the propensity of having a relatively frequent lung infection such as pneumonia. Accordingly, our approach of selecting hospitalized patients with pneumonia as a control group is intended to minimize the identification of non-specific correlates that might occur if a healthy control group was selected. Patients diagnosed with aspiration pneumonia were excluded from the study. Randomly selected 120 controls were individually matched to cases according to the year of diagnosis, in a ratio of two controls per case.

### Data collection

Information on the study variables was abstracted from the medical records of the patients of both groups. If the records indicated repeated infections with *Nocardia* or repeated hospitalizations due to nocardiosis or pneumonia, only the first episode was included.

The main dependent variable was hospitalization due to laboratory-confirmed clinical nocardiosis (see definition of cases), coded as 1; or hospitalization due to pneumonia caused by other pathogens, coded as 0.

The independent variables included demographic and clinical characteristics, which were defined based on documentation in medical records. The demographic variables included: age at the year of index hospitalization, sex, and socioeconomic rank of town of residence (following the classification of the state Central Bureau of Statistics^10^). The clinical variables included: smoking status, basic physical function (bedridden or mobile), and background illnesses (*e.g*. cancer, organ transplantation). The Charlson comorbidity index was calculated^11^.

For the 90-day period prior to the diagnosis, data were collected on hospitalizations and pharmacotherapy, including immunosuppressive and immunomodulatory treatments. These treatments included systemic and topical corticosteroids, other immunomodulatory agents (*e.g. *methotrexate and azathioprine), anti-rejection immunosuppressants (*e.g.* calcineurin inhibitors, mTOR inhibitors and mycophenolate mofetil) and anti-cancer drugs (alkylating agents, antimetabolites, anti-microtubule agents, topoisomerase inhibitors and cytotoxic antibiotics). Data on *Pneumocystis jirovecii* prophylaxis with trimethoprim/sulfamethoxazole (TMP/SMX) were collected as well.

For systemic corticosteroids, the average daily dose during the 90 days prior to the index hospitalization was documented. To facilitate comparisons of dosage, for corticosteroids other than prednisone, the prednisone equivalent dose was calculated using the standard conversion factors^12^.

As secondary objectives, we examined differences between patients with nocardiosis and patients with pneumonia at time of hospital admission in results of tests. These included vital signs, complete blood count, C-reactive protein (CRP), and albumin levels, as well as in radiological findings. We also assessed the one-year mortality after admission. These data were also collected from the medical records.

The information retrieved from hospitalization files was crosschecked against the medical records of the primary care/community HMO system and patient prescription records.

### Laboratory methods

During the study period, sputum, bronchoalveolar lavage, blood and tissue samples from infected individuals were incubated on blood and chocolate agar plates for 2–4 days. When *Nocardia* was suspected, samples were incubated also on Thayer–Martin agar plates for up to 14 days. Between 2007 and 2011, *Nocardia* identification was based on macroscopic evaluation accompanied by microscopic assessment for distinguishing features (*e.g. *pseudohyphae). These were confirmed by biochemical markers such as ortho-Nitrophenyl-β-galactoside and catalase. Since 2011, *Nocardia* was identified using matrix-assisted laser desorption/ionization time-of-flight mass spectrometer (MALDI-TOF), with a sensitivity of 95%.

### Statistical methods

Demographic and clinical variables of the patients with nocardiosis (cases) and control group (pneumonia patients) were compared using bivariate and multivariable conditional logistic regression models. Correlations between the independent variables were assessed using Spearman’s rank correlation coefficient, and highly correlated variables (correlation coefficient > 0.6) were assessed in separate models. Independent variables were selected to be included in the multivariable model based on the bivariate analysis and on prior knowledge (i.e. associated with nocardiosis such as immunosuppression treatment^1^). Independent variables were excluded from the model if they were associated with nocardiosis with *p* > 0.2, and if they yielded a change of less than 10% in the point estimate of the association between immunosuppression treatments and nocardiosis (suggesting that they were not confounders^13^). Interactions between the independent variables were assessed in the models. Collinearity between the independent variables was assessed using variance inflation factor. Matched ORs (and 95% CIs) were obtained from the conditional logistic regression models. *P* < 0.05 was considered statistically significant. Correction for multiple comparisons was conducted using the Benjamini and Hochberg false discovery rate method^14^. Sensitivity analyses were conducted to explore possible sex and other clinical related differences. The chi square test was used to assess heterogeneity in the association measures between men and women.

Data analysis was undertaken using IBM SPSS version 23 (Armonk, New York, USA) and WinPepi^15^.

### Ethical approval

The Helsinki committee of Rabin Medical Center approved the study protocol. An exempt from informed consent was given by the Helsinki committee of Rabin Medical Center due to the retrospective design of the study. We confirm that all methods were performed in accordance with the relevant guidelines and regulations.

## Results

**Figure 1:**
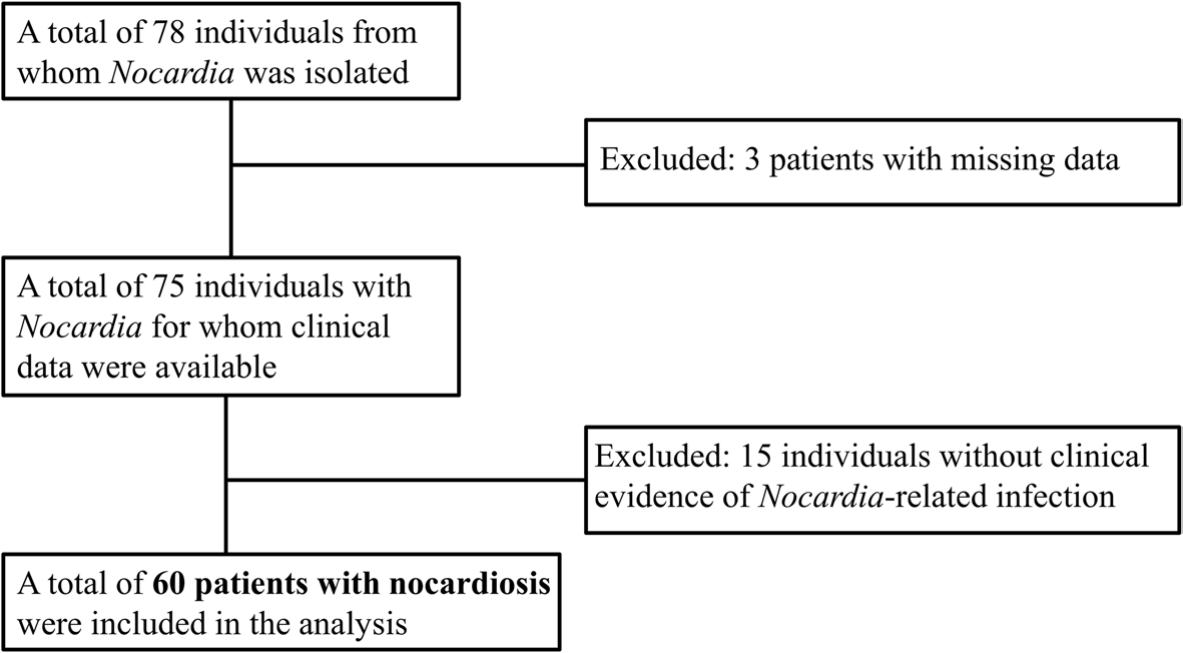
Study population selection and exclusion flowchart.

Between January 2007 and June 2018, *Nocardia* was isolated from 78 individuals treated at RMC. Of these, 18 were excluded due to either lack of sufficient data or the absence of clinical diagnosis of nocardiosis (Fig. 1). The number of patients with nocardiosis was distributed similarly along the study period (supplementary Table S1).

**Figure 2:**
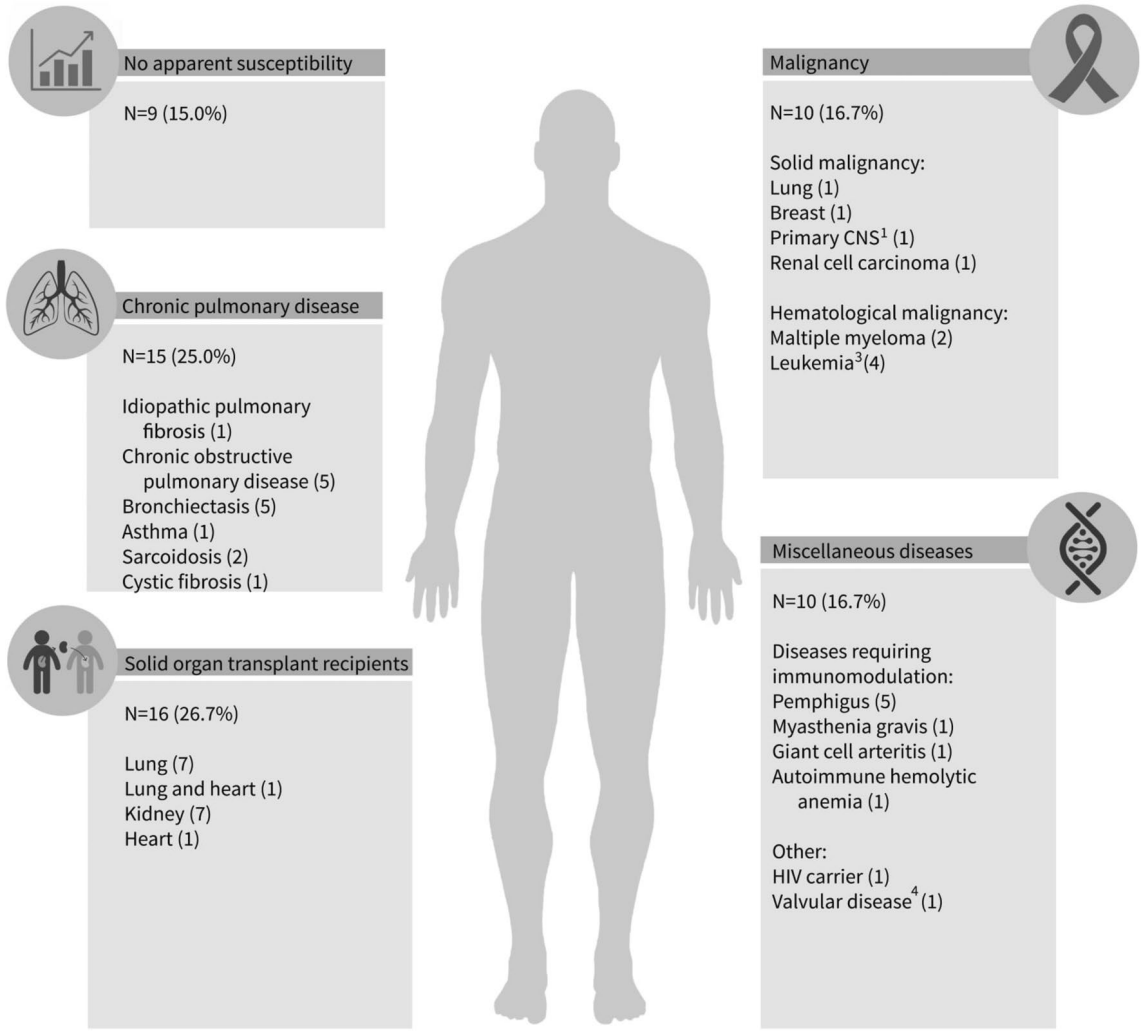
Background illnesses of the 60 patients with nocardiosis. ^1^The patient had radiological findings suspicious for glioma but she refused a biopsy; ^3^Acute lymphoblastic leukemia (1), Acute myeloid leukemia (1), Chronic lymphocytic leukemia (1), Chronic myeloid leukemia (1); ^4^The patient was diagnosed with *Nocardial* endocarditis.

The majority of the patients with nocardiosis had an immunosuppressive condition (N = 45, 75%) (see the supplementary results for the list of immune suppressive agents). Of these, the most prominent conditions were SOT (N = 16, 27%) and malignancies (N = 10, 17%). Fifteen (25%) patients had a chronic pulmonary disease, two thirds of them (N = 10) were treated with corticosteroids during the 90 days prior to the diagnosis of nocardiosis (Fig. 2).

Nocardiosis most frequently involved the lungs (N = 48, 80%). In 39 (65%) patients the lungs were the sole organ involved. Brain imaging was performed for 47 (78%) of the individuals with nocardiosis, and the infection involved the central nervous system in 10 (17%) patients. The involvement of the skin and soft tissue was found in 9 (15%) patients. One patient had *Nocardial* endocarditis that involved a prosthetic heart valve.

### Analysis of nocardiosis correlates

The mean age of patients with nocardiosis was 60.3 (15.6) years, compared to 73.8 (17.1) years in patients with pneumonia (*p* < 0.001). No significant differences were noted between the groups in sex distribution and socioeconomic status (*p* = 0.166 and *p* = 0.125, respectively) (Table 1).

**Table 1:** Characteristics of patients with nocardiosis (cases) and with pneumonia (controls).

	Nocardiosis cases N = 60	Pneumonia controls N = 120	Matched OR (95% CI)^a^	*p* value^a^	Adjusted *p* value^b^
Male sex, N (%)	29 (48.3)	74 (61.7)	0.70 (0.42–1.16)	0.166	0.216
Age (years), mean (SD)	60.3 (15.6)	73.8 (17.1)	0.98 (0.96–0.99)	< 0.001	< 0.001
Higher socioeconomic status, N (%)	42 (71.2)	100 (83.3)	0.64 (0.37–1.13)	0.125	0.203
Current smoker, N (%)	5 (8.3)	12 (10.5)	0.84 (0.34–2.10)	0.708	0.767
Bedridden, N (%)	3 (5.0)	17 (14.2)	0.42 (0.13–1.34)	0.144	0.208
Charlson comorbidity score, mean (SD)	2.1 (1.8)	2.7 (2.2)	0.90 (0.78–1.03)	0.125	0.203
Charlson comorbidity score ≥ 2, N (%)	36 (60.0)	79 (65.8)	0.85 (0.51–1.42)	0.531	0.600
Solid malignancy, N (%)	3 (5.0)	20 (16.7)	0.36 (0.11–1.15)	0.084	0.156
Hematologic malignancy, N (%)	6 (10.0)	4 (3.3)	1.89 (0.81–4.39)	0.139	0.208
Chronic pulmonary disease, N (%)	28 (46.7)	40 (33.3)	1.44 (0.87–2.39)	0.158	0.215
Solid organ transplant recipients, N (%)	16 (26.7)	3 (2.5)	3.08 (1.74–5.46)	< 0.001	< 0.001
Hospitalization in the past 90 days, N (%)	32 (53.3)	40 (33.3)	1.71 (1.03–2.85)	0.037	0.074
Hospitalization duration (days), median (IQR)	7 (12)	4 (4)	1.03 (1.00–1.07)	0.069	0.020
Systemic immunosuppression, N (%)	44 (73.3)	23 (19.2)	4.64 (2.62–8.22)	< 0.001	< 0.001
≥ 2 immunosuppressive agent, N (%)	21 (35.0)	9 (7.5)	2.69 (1.58–4.58)	< 0.001	< 0.001
Topical corticosteroids^c^, N (%)	13 (21.7)	7 (5.8)	2.21 (1.20–4.09)	0.011	0.028
Concurrent systemic and topical corticosteroids, N (%)	10 (16.7)	0	3.43 (1.74–6.74)	< 0.001	< 0.001
Topical without systemic corticosteroids, N (%)	3 (5.0)	7 (5.8)	0.89 (0.28–2.86)	0.851	0.851
Systemic corticosteroids, N (%)	43 (71.7)	16 (13.3)	5.19 (2.96–9.09)	< 0.001	< 0.001
Prednisone equivalent dose^d^ (mg), mean (SD)	33.8 (30.8)	30.2 (42.4)	1.00 (0.99–1.01)	0.850	0.851
Prednisone equivalent dose^d^ > 10 mg/d, N(%)	34 (79.1)	8 (50.0)	1.53 (0.73–3.19)	0.257	0.303
Preventive TMP/SMX treatment, N (%)	11 (18.3)	1 (0.8)	3.14 (1.63–6.04)	0.001	0.003

*CI* confidence interval, *IQR* interquartile range, *OR* odds ration, *SD* standard deviation, *TMP/SMX* trimethoprim and sulfamethoxazole.

^a^Calculated using conditional logistic regression models; ^b^Calculated using Benjamini and Hochberg false discovery rate method; ^c^Either inhaled corticosteroids or dermal preparations, regardless of systemic use; ^d^Daily prednisone dosage (in milligrams). Whenever dexamethasone was used, dosage was converted to the equivalent prednisone dosage.

Mean scores on the Charlson comorbidity index did not differ significantly between patients with nocardiosis and those with pneumonia (*p* = 0.125). The proportion of patients with SOTR was higher in the case than control group: 27% versus 3%, *p* < 0.001, while the proportion with solid malignancies was lower: 5% versus 17%, *p* = 0.084. A higher proportion in the nocardiosis group was hospitalized during the 90 days prior to the diagnosis of nocardiosis than the control group: 53% versus 33%, *p* = 0.037.

Compared to those with pneumonia, the patients with nocardiosis were more often treated with immunosuppressive and other immunosuppression conferring agents (*i.e. *immunomodulatory agents, anti-rejection immunosuppressants and anti-cancer drugs), and more often received prophylactic treatment with TMP/SMX (18% vs. 1%, *p* = 0.001), systemic immunosuppression agents (73% vs. 19%, *p* < 0.001) or more than one immunosuppressive agent (35% vs. 8%, *p* < 0.001). The use of corticosteroids was documented among 72% of patients with nocardiosis versus 13% of those with pneumonia (*p* < 0.001). Among individuals administered systemic corticosteroids, the dosage of corticosteroids did not differ significantly between the case and control groups (*p* = 0.850). The adjusted *p* values calculated for multiple comparisons yielded similar results.

### Multivariable analysis

Before conducting a multivariable analysis, correlations between the independent variables were assessed. Mostly weak or non-statistically significant correlations were found.

A multivariable conditional logistic regression model that included all the patients with nocardiosis showed that pharmacotherapy with any immunosuppressive agent was strongly positively associated with nocardiosis: matched OR 4.40 (95% CI 2.25–8.62), *p* < 0.001. In this model, the inverse association between age and nocardiosis remained significant, while the associations of sex, Charlson comorbidity index, SOT and hospitalizations during the 90 days prior to nocardiosis were not statistically significant (Table 2).

**Table 2:** Multivariable conditional logistic regression model of the risk factors for nocardiosis.

	Adjusted matched OR (95% CI)^a^	*p* value^a^
Any immunosuppressive agents	4.40 (2.25–8.62)	< 0.001
Age (per decade)	0.83 (0.71–0.98)	0.022
Male sex	1.39 (0.83–2.32)	0.213
Charlson comorbidity score	0.89 (0.77–1.03)	0.125
Solid organ transplant recipient	0.97 (0.49–1.92)	0.918
Hospitalization in the past 90 days	0.96 (0.55–1.67)	0.873

*CI* confidence interval, *OR* odds ratio.

^a^Obtained from conditional logistic regression models adjusted for the variables presented in the table.

Another model included treatment with corticosteroids as the main independent variable showed a strong positive association between treatment of corticosteroids and nocardiosis: matched OR 4.69 (95% CI 2.45–8.99), *p* < 0.001, while adjusting for the aforementioned confounders (Table 3).

**Table 3:** Adjusted associations between treatment with corticosteroids and nocardiosis: multivariable conditional logistic regression models.

	N	Adjusted matched OR (95% CI)^a,b^	*p* value ^b^
Total study population	180	4.69 (2.45–8.99)	< 0.001
Amongst males	103	7.61 (2.74–22.16)	< 0.001
Amongst females	77	3.29 (1.27–8.52)	0.014
Amongst patients with chronic pulmonary disease	68	5.74 (1.92–17.15)	0.002
Systemic corticosteroids in patients with pulmonary nocardiosis^c^	144	5.90 (2.75–12.66)	< 0.001

*CI* confidence interval, *OR* odds ratio.

^a^Adjusted for age, sex, Charlson score, solid organ transplantation, and hospitalization during the 90 days prior to the diagnosis; ^b^Obtained from conditional logistic regression models; ^c^Systemic corticosteroids as a risk factor for nocardiosis involving the lungs (only pulmonary involved nocardiosis cases were included in this analysis).

Stratifying the analysis by sex showed a stronger association between treatment with corticosteroids and nocardiosis among males (matched OR 7.61 [95% CI 2.74–22.16, *p* < 0.001]) than among females (matched OR 3.29 [95% CI 1.27–8.52], *p* = 0.014) (Table 3); however, the chi square test for heterogeneity was not significant (chi square 1.35*, p* = 0.245). Limiting the analysis to patients with chronic pulmonary disease showed that the association between treatment with corticosteroids and nocardiosis might be stronger than in the general sample of nocardiosis patients: matched OR 5.74 (95% CI 1.92–17.15, *p* = 0.002), but the CIs were overlapping. Similar results were obtained when the analysis was limited to patients with pulmonary nocardiosis (Table 3).

The independent role of SOT in nocardiosis was evaluated in models that included SOT as the main independent variable. In this analysis, the positive association between SOT and nocardiosis was attenuated following adjustment for demographic and clinical factors, and became non-significant following adjustment to the dosage of consumed systemic corticosteroids (Table 4).

**Table 4:** Solid organ transplantation and the risk for nocardiosis: multivariable conditional logistic regression models.

	Matched OR (95% CI)^a^	*p* value^a^
Unadjusted	3.08 (1.74–5.46)	< 0.001
Model 1^b^	2.12 (1.14–3.94)	0.018
Model 2^c^	1.91 (1.00–3.65)	0.052
Model 3^d^	1.41 (0.59–3.40)	0.440

*CI* confidence interval, *OR* odds ratio.

^a^Obtained from conditional logistic regression models; ^b^Adjusted for age and sex; ^c^Adjusted for age, sex, Charlson score, and hospitalization during the 90 days prior to the diagnosis; ^d^Adjusted for age, sex, Charlson score, hospitalization during the 90 days prior to the diagnosis, and the daily prednisone dosage in milligrams (whenever dexamethasone was used, dosage was converted to the equivalent prednisone dosage).

### Radiological characteristics

The majority of patients with pulmonary nocardiosis (N = 48) had either solitary infiltrate (N = 17, 35%) or bilateral infiltrates (N = 11, 23%). Solitary nodule (N = 1, 2%) or multiple nodules (N = 5, 10%) were also observed, and 4 patients (8%) had both infiltrates and nodules. Additional 4 (8%) patients had a pulmonary abscess. Six (13%) patients had other radiographic presentations, such as pleural effusion or dissolution of the surgical anastomoses.

Among 111/120 (93%) of patients with pneumonia (control group), a pulmonary infiltrate was evident on chest radiogram on admission. Among the remaining nine individuals, pneumonia was diagnosed based on clinical signs and symptoms (i.e. fever, respiratory symptoms, elevated markers of inflammation and compatible auscultator findings). Bilateral diffuse pneumonia was evident in 14/111 (13%) of pneumonia controls, whereas single side pneumonia was evident in 97/111 (87%) of them.

### Vital signs and blood parameters at admission

Among patients with nocardiosis, the mean saturation was significantly higher, and the proportion with dyspnea was lower, than among patients with pneumonia. The mean hemoglobin and albumin levels were significantly lower in patients with nocardiosis than in patients with pneumonia. No significant difference was found between the groups in CRP and in counts of white blood cells and platelets (Table 5).

**Table 5:** Vital signs and blood parameters of individuals with nocardiosis and with pneumonia, measured at time of hospital admission.

Variable	Nocardiosis cases N = 60	Pneumonia controls N = 120	OR^a^	95% CI^a^	*p* value^a^
Systolic BP, mean (SD)	122.5 (25.1)	121.1 (23.7)	1.00	0.99–1.02	0.724
Diastolic BP, mean (SD)	70.5 (12.4)	65.7 (13.8)	1.03	1.00–1.05	0.037
Pulse (BPM), mean (SD)	87.8 (15.4)	87.9 (20.4)	1.00	0.98–1.02	0.964
Saturation (%), mean (SD)	95.1 (4.1)	90.7 (7.0)	1.20	1.10–1.32	< 0.001
Dyspnea N, (%)	21 (41.2)	72 (62.1)	0.43	0.22–0.84	0.013
Hemoglobin (g/dL), mean (S)	11.2 (1.9)	12.1 (2.1)	0.80	0.68–0.94	0.007
White blood count, mean (SD)	14.6 (20.5)	13.5 (8.5)	1.01	0.98–1.03	0.608
Neutrophils (%), mean (SD)	78.4 (15.8)	79.2 (14.2)	1.00	0.98–1.02	0.741
Platelets, mean (SD)	251.8 (103.5)	246.7 (110.9)	1.00	1.00–1.00	0.764
Albumin (g/dL), mean (SD)	3.3 (0.6)	3.5 (0.6)	0.50	0.28–0.89	0.018
C-reactive protein, mean (SD)	11.6 (10.2)	14.2 (11.1)	0.98	0.94–1.02	0.274

^a^Obtained from conditional unadjusted logistic regression models.

*BP* blood pressure, *BPM* beats per minute, *CI* confidence interval, *OR* odds ratio, *SD* standard deviation.

Blood and sputum cultures from the control group (patients with pneumonia) were positive in 14/120 (12%), with *Streptococcus pneumoniae* being the leading pathogen (29%) isolated from the blood.

### Overall survival

There was no significant difference in mortality one year after hospitalization between patients with nocardiosis and the pneumonia controls: 33% and 38% respectively, *p* = 0.623.

## Discussion

We compared the correlates between patients with nocardiosis and hospitalized patients with pneumonia, thus enabling the assessment of clinical correlates of nocardiosis beyond the propensity to develop pulmonary bacterial infection in general.

The main finding of the current study is that treatment with any immunosuppressive agent and particularly with systemic corticosteroid therapy was strongly related to an increased risk for nocardiosis, independent of demographic characteristics and other recognized risk factors for nocardiosis. Our finding regarding the association between corticosteroid therapy and nocardiosis is similar to that reported by Coussement *et al*.^1^. In their multicenter study of SOTR, the OR for nocardiosis was 1.12 per each milligram of corticosteroids. In the current study, the association between corticosteroid therapy and nocardiosis surmounted the association between a history of SOT and nocardiosis. This suggests that the history of SOT is merely a mediator variable for corticosteroid therapy rather than an independent risk factor for nocardiosis. Indeed, when the use of systemic corticosteroid therapy was included in the regression model, the association between SOT and nocardiosis was not significant. Coussement *et al*.found that high through levels of calcineurin inhibitors increased the risk for nocardiosis substantially, compared with corticosteroid therapy. As there were only 16 (27%) SOTR in our cohort, statistical power was lacking to evaluate calcineurin inhibitor as a risk factor for nocardiosis amongst SOTR.

The point estimate for the measure of association of systemic corticosteroid therapy with nocardiosis was somewhat higher amongst individuals with chronic pulmonary disease than in the cohort as a whole. *Nocardia* is an intracellular bacteria^16^ and the host's immune reaction against infection broadly relies on macrophage activity^17^. In contrast to nocardiosis, pneumonia in hospitalized patients is usually caused by typical, extracellular bacteria^18^. Individuals with chronic pulmonary diseases were found to have a higher rate of macrophage dysfunction^19^. It is therefore assumed that the macrophage activity of individuals with chronic pulmonary diseases treated with corticosteroids is particularly decreased, exposing them to a higher risk for nocardiosis. Corticosteroid therapy particularly damages the alveolar macrophages^20^. This can explain the increased effect of such treatment on the risk of developing pulmonary nocardiosis.

Systemic corticosteroid therapy is known to increase the risk for respiratory infections in general, including pneumonia^21,22^. In the current study, the inclusion of patients with pneumonia was not restricted to those with isolated bacterial pathogens, and it is likely that some of the patients in the control group had viral pneumonia. However, in comparing patients in the nocardiosis and the control groups, we were able to distinguish correlates that were associated with nocardiosis relative to ordinary pneumonia.

Overall, 60 patients with clinically and laboratory-confirmed nocardiosis were identified for the 12-year period. Although different methods for identifying *Nocardia* were used in the earlier and later periods, the average yearly number of patients with nocardiosis did not differ significantly over the study period. We therefore assume that nocardiosis incidence at our center was not affected by diagnostics. Previous studies from two other tertiary hospitals in Israel reported 39–53 cases of nocardiosis during a similar time interval, about one decade ago^23,24^. The higher number of cases observed in our study might be due to differences in case definitions across the studies. However, it also might reflect an increase in the incidence of nocardiosis. Advances in medical technologies have led to an increased prevalence of individuals living with transplanted organs^25^ or with chronic pulmonary diseases^26^, thus might have resulted in a larger population susceptible to nocardiosis. RMC is a tertiary hospital in the central district of Israel. In addition to serving residents of the central district, RMC is a referral hospital that serves patients from around the country. Moreover, due to the additional tertiary hospitals in the region, the exact size of the population served by RMC cannot be estimated accurately. Accordingly, in the absence of a concrete denominator, we could not estimate the nocardiosis incidence rate. However, for a single center, the number of patients with nocardiosis in our series was higher than reported over a similar period in France^27^. This might be due to differences in patients' mix and case definitions.

While former reports showed that males comprised more than half the patients with nocardiosis^1,27–29^, males comprised only 48% of our patients with nocardiosis. We found no significant difference in the distribution of sex between the groups. However, the association between corticosteroid therapy and nocardiosis was stronger in males. Nonetheless, the heterogeneity test was not significant, likely due to the small sample size. An underestimation of the realistic effect of this interaction is possible, considering the lower adherence rates of males to pharmacotherapy in general^30^, and to immunomodulatory therapy in particular^31^. However, previous reports on the susceptibility to opportunistic infections in the setting of corticosteroid therapy did not show a difference between the sexes^32,33^. As corticosteroid use may reflect the severity of the underlying disease, the susceptibility of corticosteroid-treated males to nocardiosis may merely be a surrogate for their poorer physical condition, exposing them to nocardiosis. In our study, most of the chronic lung patients had either chronic obstructive pulmonary disease or bronchiectasis, diseases that require systemic corticosteroid therapy for acute exacerbations^34,35^, and which carry a poorer prognosis in males^36,37^. Further research is required to elucidate this issue.

The current study had several limitations, particularly the retrospective design. Differences in the recording of medical information might have existed between physicians; and the accuracy regarding some of the study variables, such as pharmacotherapy and dosages, might not be optimal. To increase the validity of the information retrieved from hospitalization files, data were crosschecked against other medical charts, such as the HMO systems and patients' prescription records. Medical data, such as physicians' prescriptions and medication purchasing are continuously and automatically recorded for all insured members. Thus, differential misclassification of the main study variables, such as immunosuppressive pharmacotherapy, is unlikely. Moreover, since the study sample comprised hospitalized patients with a relatively high comorbidity burden, such information bias, if existent, would likely be non-differential. Notably, since nocardiosis is a relatively rare infection, the total number of cases was too small to yield statistical power for assessing risk factors in specific sub-populations.

As a single center study, a selection bias should be considered. However, it is unlikely that patients with nocardiosis at RMC differ from patients diagnosed at other tertiary hospitals. Moreover, the selection of nocardiosis patients and control patients with pneumonia from the same hospital mitigates differences in referral patterns between the groups. As all patients with nocardiosis at RMC during the study period were included in the study, selection bias of the cases is unlikely. We combined all the patients with nocardiosis as one group, though their underlying diseases varied (as demonstrated by the inclusion of SOT and pemphigus). This approach might have led to the "dilution" of a given factor that specifically characterized a subgroup of patients. This limitation was dictated by the small number of patients with nocardiosis, and the even smaller numbers of important subgroups such as SOT. Consequently, addressing the effect of anticalcineurin inhibitor type or serum level on the risk of developing nocardiosis was limited. Future large multicenter studies are needed to address the specific correlates and risk factors of nocardiosis, and especially to investigate subgroups of patients with underlying diseases.

Selection bias of the control group was minimized by random sampling from the hospitalization registry. The control group in our study comprised hospitalized patients with pneumonia. It might be argued that this is not the optimal control group, and having healthy persons as controls is warranted. However, since nocardiosis is an opportunistic infection that mostly affects the lungs, our aim was to identify specific correlates of nocardiosis. The selection of healthy persons as controls could have resulted in identifying correlates of disease and hospitalization in general, or inflated measures of association. Therefore, patients with pneumonia were considered the most appropriate control group to address the study goal.

To our knowledge, this is the first study that assessed multiple correlates of nocardiosis. Corticosteroid therapy was strongly positively related to nocardiosis. The effect size was robust and varied amongst sub-populations; it was remarkably strong for pulmonary nocardiosis. Males and patients with chronic pulmonary diseases might be at a greater risk for nocardiosis when treated with corticosteroids. Our findings have clinical implications in the identification of patients at risk for nocardiosis and can guide clinicians' decision-making on immunomodulatory treatment.

## Supplementary information

Supplementary information

## Data Availability

The datasets generated during and/or analyzed during the current study are available from the corresponding author on reasonable request.
