# Supplementary material for: Clinical correlates of nocardiosis
Source: Sci Rep. 2020 Aug 31;10:14272. doi: 10.1038/s41598-020-71214-4 (PMC7459281; doi:10.1038/s41598-020-71214-4)
Supplement: Supplementary file 1 — Supplementary information [file 41598_2020_71214_MOESM1_ESM.pdf]

Clinical correlates of nocardiosis

Ili Margalit[*1,2], Elad Goldberg[1,2], Yaara Ben Ari[2], Haim Ben-Zvi[3], Yael Shostak[4], Ilan Krause[1,2], Khitam Muhsen[5]

[1]Department of Internal Medicine F-Recanati, Rabin Medical Center, Beilinson Hospital, Petah Tikva, Israel.

[2]Sackler Faculty of Medicine, Tel Aviv University, Ramat Aviv, Tel Aviv, Israel.

[3]Microbiology Laboratory, Rabin Medical Center, Beilinson Hospital, Petah-Tikva, Israel.

[4]Pulmonary Institute and Department of Internal Medicine D, Rabin Medical Center, Beilinson Hospital, Petach Tikva, Israel.

[5]Department of Epidemiology and Preventive Medicine, School of Public Health, Sackler Faculty of Medicine, Tel Aviv University, Ramat Aviv, Tel Aviv, Israel.

Supplementary methods 1

List of the World Health Organization International Classification of Diseases 9th Revision (ICD-9) codes considered as diagnosis of *pneumonia*, for the purpose of the current study

ICD-9 code 486: Pneumonia

BACTERIAL PNEUMONIA, UNSPECIFIED

BRONCHOPNEUMONIA, ORGANISM UNSPECIFIED

OTHER BACTERIAL PNEUMONIA

PNEUMONIA

PNEUMONIA DUE TO OTHER SPECIFIED BACTERIA

PNEUMONIA DUE TO OTHER SPECIFIED ORGANISM

PNEUMONIA, COMMUNITY ACQUIRED

PNEUMONIA, HOSPITAL ACQUIRED

PNEUMONIA, ORGANISM UNSPECIFIED

Note: patients diagnosed with *aspiration pneumonia* were not included as controls.

Supplementary results

List of immune suppressive agents administered to patients in the current study during the 90 days prior to their diagnosis

Systemic corticosteroid therapy: dexamethasone and prednisone

Other immunomodulatory agents: azathioprine and methotrexate

Anti-rejection agents: ciclosporin, mycophenolate mofetil, tacrolimus

Anti-cancer agents: asparaginase, carboplatin, cyclophosphamide, cytarabine, daratumumab, docetaxel, doxorubicin, etoposide, gemcitabine, isophosphamide, lenalidomide, nivolumab, olaratumab, pablociclib, vincristine.

Supplementary Table S1: The distribution of nocardiosis cases by calendar year (2007-2018)

Year of diagnosis	Number of nocardiosis cases	Proportion of cases of all years
2007	5	0.08
2008	6	0.10
2009	6	0.10
2010	6	0.10
2011	4	0.07
2012	5	0.08
2013	3	0.05
2014	5	0.08
2015	7	0.12
2016	4	0.07
2017	2	0.03
2018	7	0.12
Total	60	1.00
